# Effect of progesterone administration on tissue mast cell population and histamine content in mice uterus after ovulation induction

**DOI:** 10.5935/1518-0557.20230004

**Published:** 2023

**Authors:** Negar Azadi, Nasim Beigi Boroujeni, Maryam Hormozi, Leila Narimani, Majid Tavafi, Khatereh Anbari, Mandana Beigi Boroujeni

**Affiliations:** 1 Razi Herbal Medicines Research Center, School of Medicine, Lorestan University of Medical Sciences, Khorramabad, Iran; 2 Social Determinants of Health Research Center, School of Medicine, Lorestan University of Medical Sciences, Khorramabad, Iran

**Keywords:** progesterone, histamine, ovulation, assisted reproduction

## Abstract

**Objective:**

Mast cell population and histamine affect on blastocyst implantation. This study aimed to evaluate the effects of progesterone administration after induction of ovulation on the uterine tissue mast cell population and histamine content in mice.

**Methods:**

We ran an experimental study on three groups of mice; control group, ovulation induction (induction group), and ovulation induction along with progesterone administration (progesterone group). Mast cells were counted using toluidine blue staining, and the histamine level was measured through spectrophotometry.

**Results:**

According to the analysis of variance (ANOVA), there was no difference in mast cell population in endometrium (*p*=0.138) nor in myometrium (*p*=0.611). The ratio of mast cells in the myometrium per endometrium increased in the progesterone group in comparison to the control group based on a generalized linear model (*p*=0.041). The uterine histamine level was different between the groups, based on the ANOVA (*p*=0.039), in which the progesterone group had lower amounts of histamine.

**Conclusions:**

Progesterone administration after ovulation induction did not decrease the number of endometrial mast cells and could have increased the ratio of myometrium mast cells per endometrium mast cell. The histamine level *in uterus* decreased by the administration of progesterone in the ovulation-induced mice.

## INTRODUCTION

Ovarian stimulation is used in infertility treatments and results in simultaneous growth of several follicles during a one month cycle (Gad *et al*., 2011). The uterus is made up of endometrium, myometrium and perimetrium. The endometrium is a unique tissue that undergoes proliferation, differentiation, destruction and repair. Such processes are necessary for embryo implantation. These cycles are affected by sexual steroid hormones (King & Critchley, 2010).

Mast cells (mastocytes) are a part of the innate immune system, stemming from the bone marrow. The major source of tissue histamine is the mast cell and they mature in their targeted tissues (Witkowski *et al*., 2015). The role histamine released from the uterine mast cells is notable upon implantation, pregnancy and delivery (King & Critchley, 2010). Histamine derived from the uterus is known as a major regulatory agent playing a role in implantation through the induction of vascular permeability and decidualization (Jensen *et al*., 2010). Female sexual hormones affect mast cell function. Estradiol and progesterone result in mast cell maturation, especially maturation of uterine mast cells (King & Critchley, 2010). These hormones also affect mast cell activation (Zierau *et al*., 2012). Reduction of mast cells upon implantation is associated with increased estrogen, believing that implantation time decidualization is regulated by the histamine released from mast cells (Harvey, 1964). It has been reported that mast cells in humans and rodents are necessary for ovulation, implantation of blastocysts and placentation. In addition, mast cells support decidual repair and uterine contractions (Meyer *et al*., 2018). Woidacki *et al*. (2013) mentioned that mast cells had receptors for estradiol and progesterone. Hamouzova *et al.* (2016) mentioned that the most number of mast cells was seen when estradiol levels were at their upper limit (Hamouzova *et al*., 2016). Noor *et al.* (2010) mentioned that histamine affects implantation, ovulation, regulation of secretions of placental blood and myometrium contraction.

Sexual hormones balance change during ovulation induction, and considering the roles of mast cells and histamine in the implantation process, the present study aimed to investigate the effects of progesterone administration after ovulation induction on the uterine tissue mast cell population and histamine content in mice.

## MATERIALS AND METHODS

### Study design and groups

We ran this experimental study on 15 NMRI mice aged 6-8 weeks. The animals were kept for two estrus cycles under standard condition. Then, they were broken down into three groups, including the control group, ovulation induction group (called induction group), and a group for progesterone administration after ovulation induction (called progesterone group). Concerning the control group (n=5), pseudo pregnancy was performed through vaginal swabbing, and after 3.5 days the animals were slaughtered for study. For induction group (n=5), a single dose of 10 IU human menopausal gonadotropin (hMG) hormone was injected intraperitoneally (IP), followed by a single dose of 10 IU human chorionic gonadotropin (hCG) hormone IP injection after 48 hours to induce ovulation. Then pseudo pregnancy was performed and after 3.5 days the animals were slaughtered for the study. Concerning the progesterone group (n=5), the process in the induction group was performed followed by daily administration of 1 mg subcutaneous (SC) progesterone for up to 3.5 days (Narimani *et al*., 2020). Ethical guidelines for working with laboratory animals were followed and the protocol of this study was approved in the ethics committee of Lorestan University of Medical Sciences under registration number IR.LUMS.REC.1399.300.

### Tissue study and mast cell count

In order to take samples, the animals were slaughtered by neck dislocation after general anesthesia. One uterus horn was used for tissue study and the other horn was used for histamine analysis. For tissue study, the mid one-third of uterus horns were separated and fixed in formalin solution for 24 to 48 hours. The paraffin processing was performed manually. After tissue processing, the samples were embedded horizontally in paraffin in order to make cross-sections from paraffin blocks. Then 5µm serial sections were prepared in which each 5 consecutive sections were used for one staining method as sections 1 - 5 for hematoxylin and eosin (H&E), sections 6 - 10 for periodic acid Schiff (PAS), sections 11 - 15 for toluidine blue staining and this sequence was repeated.

Toluidine blue staining was performed to stain mast cells; H&E and PAS staining were used for qualitative morphological study of decidualization. After dewaxing, the samples were stained, and then they were dehydrated in ascending alcohol concentrations, clear in xylene and mounted with a cover glass. For H&E staining, the samples were put in hematoxylin Harris that we prepared *in house* according to Suvarna *et al.* (2018) from hematoxylin crystal (No. 1.04302.0025, Merck, Germany), for 5 min, and after we washed in water, acid alcohol, water again, lithium carbonate 1% (No. 1.05680.0520, Merck, Germany); water again; then the samples were put in eosin - prepared from dry eosin (No. 1.15935.0025, Merck, Germany) according to Suvarna *et al.* (2018) for 2 min. PAS staining was performed *in house* according to Suvarna *et al*. (2013). For toluidine blue staining, 1 g toluidine powder (No. 1.15930.0025, Merck, Germany) was mixed with 20 ml alcohol 95%, 80 ml distilled water and 1 ml glacial acetic acid. The concentration of toluidine blue was 1% with Ph 1.0 - according to the literature (Karaca *et al*., 2008; Liu *et al*., 2012).

To count mast cells per area, 10 sections were studied in different fields as the morphometric method of using a square frame 8.5×8.5 cm with magnification ×400 a under LEICA microscope (DM500, ICC50 HD, Germany), using Leica Applications Suit LAS ES Version 3.4.0 (Leica microsystems limited, 2016) software. The count process was performed in the endometrium and the myometrium. The count was done per area (later in the paper this variable is called mast cell count instead of mast cell count per area). The morphometric protocol used was that discussed by Gundersen *et al.* (1988).

### Tissue histamine level

After separating the samples and clearing fat and extra tissues, they were kept temporarily at -70ºC until histamine analysis. The samples were thawed and homogenized as follows: the samples were added four folds (volume wise) of sodium chloride 0.85% (1:4 v/v) in homogenizer (ULTRA TURRAX, IKA-T18 basic, Germany). Then the samples were passed through a filter. Thereafter, the samples were centrifuged at 12000 rpm for 10 min. We used a cold reagent: 1.5 ml of sulphanilic acid (0.9% w/v) (No. 8.22338.1000, Merck, Germany) in 4% hydrochloric acid (No. 1.00317.2500, Merck, Germany), plus 1.5 ml of sodium nitrite 5% w/v poured into a 50 ml volumetric container and kept in an ice bath for 5 minutes. Some 6 ml of 5% sodium nitrite was added and then cooled to 25 ml with cooled distilled water and kept in an ice bath for 15 minutes. Measurement method: 5 ml of sodium carbonate 1.1% (NO.1.06398.1000, Merck, Germany) was slowly mixed with 2 ml of cold reagent and then 1 ml of the homogenized sample was added to it, after 5 minutes of absorption at 496 wavelength, it was read by a spectrophotometer. Distilled water was used as a blank (histamine was prepared as standard from 0-100µg/ml and measured). We used a colorimetric method to evaluate the histamine levels using a spectrophotometer (Cecil instrument CE2020, Serial Number 93183) at a wavelength of 496 nm. Histamine was used as the standard from 0 - 100 µg. Then the histamine level was reported at the unit of µg/g-tissue.

### Statistical analysis

The sample size was calculated based on the “law of diminishing returns”, proposed by Charan & Kantharia (2013) for animal studies. Accordingly, we had five samples for each of the three groups, which resulted in the acceptable range of 10 to 20 degrees of freedom for variance analysis (ANOVA) (Arifin & Zahiruddin, 2017; Charan & Kantharia, 2013). Therefore, we used the one way ANOVA with Tukey post hoc test to compare the variability of the means between the groups of study. When non-significant, we used the generalized linear modeling with Poisson family for such cases. Non-parametric tests did not have enough power for this sample size. *P*=0.05 was considered as the significance level. We used the software packages SPSS24 (IBM, US) and Stata14 (Stata Corp. LLC, US) to analyze and plot the data.

## RESULTS

**Figure 1:**
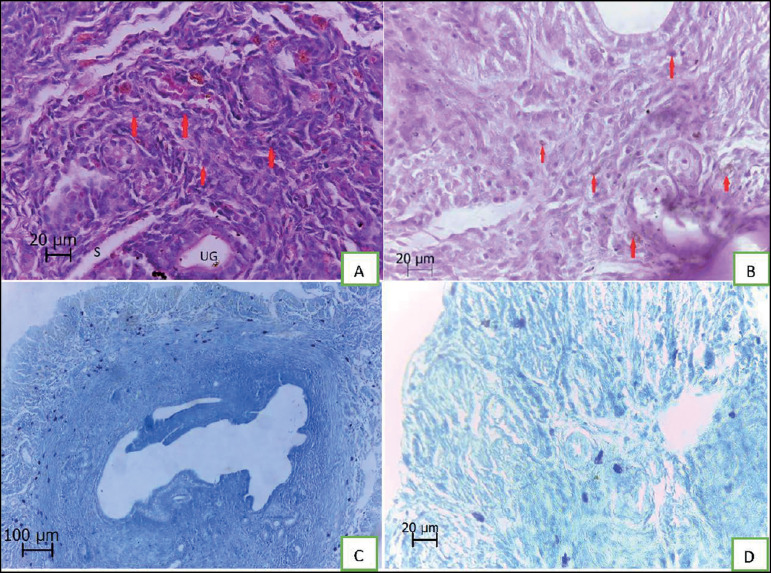
Morphology of uterus samples. Micrographs A: UG indicates uterine glands, S indicates sinusoid and the arrows indicate decidual cells (H&E, ×400). Micrographs B: decidualization of endometrium and the arrowed cells indicate large granular inflammatory cells (PAS, ×400). Micrographs C and D: The dark blue cells indicate mast cells (toluidine blue, ×100 [C] and ×400 [D]). Distribution of mast cells is seen that most of them are in myometrium.

**Figure 2:**
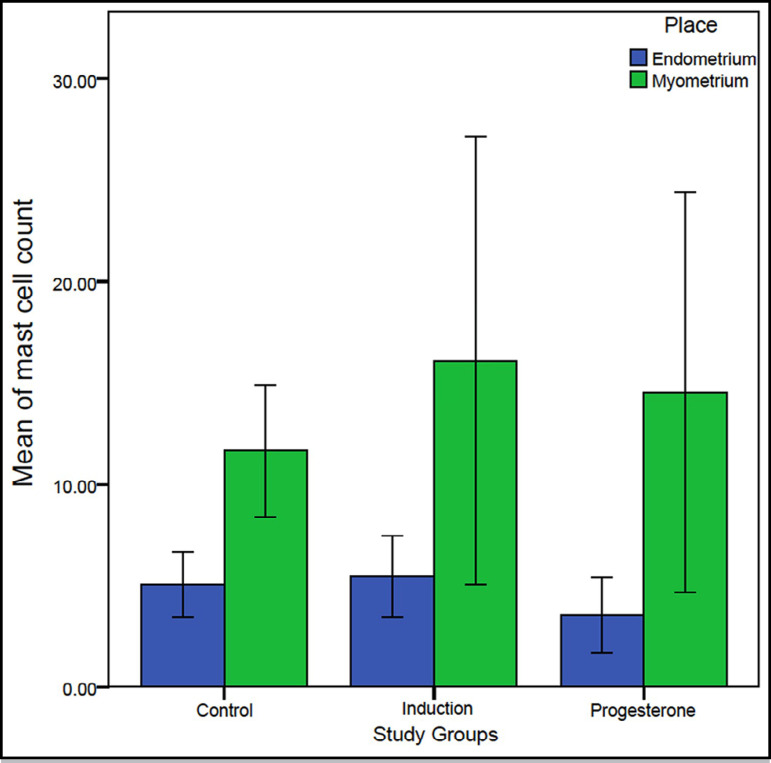
The mean of mast cell count per area among the groups and places. No significant difference was observed (ANOVA). The error bars indicate 95% CI.

Qualitative study of the tissues showed successful decidualization in progesterone group (Figure 1, A and B). The mast cells were counted in endometrium and myometrium (Figure 1, C and D). The means of mast cell count in the groups of study were compared. According to the ANOVA, the differences of the means were not significantly greater than the differences existed within the groups neither in endometrium (*p*=0.138) nor in myometrium (*p*=0.611) (Table 1, Figure 2).

**Table 1:** Comparison of mast cell count between the groups of study in endometrium, myometrium and myometrium per endometrium ratio.

Place	Groups/mean (SD)			ANOVA
	Control	Induction	Progesterone	*p* value
Endometrium	5.06 (1.30)	5.46 (1.62)	3.55 (1.48)	0.138
Myometrium	11.65 (2.63)	16.09 (8.87)	14.53 (7.94)	0.611
Myo/Endo ratio	2.41 (1.47)	2.90 (1.68)	4.94 (0.89)	0.151

Myo/Endo: myometrium per endometrium.

* Significant at 0.05.

**Figure 3:**
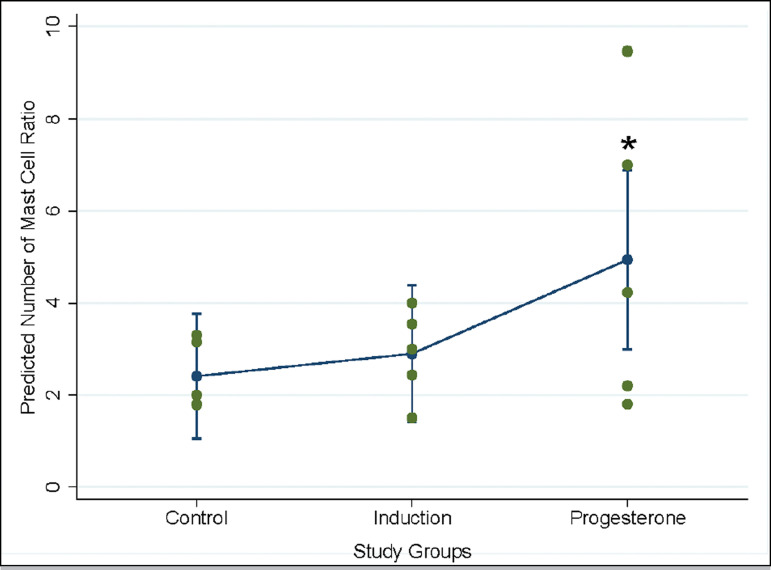
Poisson regression for prediction of mast cell count ratio in myometrium per endometrium (the plot is marginal post estimation output of the regression, Stata14). The error bars indicate 95% CI. * Significant difference considering the control group as the base.

According to the increasing mast cell count in myometrium (against control group) along with decreasing the mast cell count in endometrium (against control group), the new variable “myo/endo ratio” was generated indicating the ratio of mast cell count in myometrium per mast cell count in endometrium. Nevertheless, the means of this ratio were not significantly different between the groups of study (*p*=0.151) (Table 1). Since general linear model (i.e. ANOVA) could not predict the outcome, generalized linear model (here Poisson regression) was conducted. Accordingly, progesterone group showed a significant higher myo/endo ratio in comparison to the control group (incidence rate ratio [IRR] =2.051, *p*=0.041) (Table 2, Figure 3). Poisson regression was also performed for mast cell count in endometrium and myometrium, but no significant result was observed (not shown).

**Table 2:** Estimation of mast cell myo/endo ratio based on general and generalized linear model.

Group	General linear model (ANOVA)		Generalized linear model (Poisson)	
	*Beta* coefficient	*p* value	IRR	*p* value
Control	Reference		Reference	
Induction	0.489	0.708	1.203	0.636
Progesterone	2.532	0.070^#^	2.051	0.041^*^
Constant	2.408	0.020	2.408 (e form)	0.002
R square	0.270		0.083 (pseudo)	

* Significant at 0.05.

# Significant at 0.1.

Uterine histamine level was compared between the groups. According to the ANOVA, the differences of the means were significantly greater than the differences existed within the groups (*p*=0.039) in which progesterone group showed lower amounts of histamine in comparison to other groups based on Tukey post hoc test (Table 3).

**Table 3:** Comparison of uterus histamine level (µg/g-tissue) between the groups study.

Group	Mean (SD)	ANOVA	Post hoc
		*p* value	Significance
Control	8.79 (2.13)	0.039^*^	No
Induction	8.56 (1.51)		No
Progesterone	5.49 (1.32)		Yes ^#^

* Significant at 0.05.

# *Versus* both induction and control groups.

## DISCUSSION

Induction of ovulation is a process commonly used in management of infertility. Progesterone administration is a way to improve success rate of implantation. Animal studies are performed to find the mechanisms of effect for progesterone. The present study was performed to show the effect of progesterone on endometrium and myometrium mast cell population and uterus histamine level after induction of ovulation in a mice model.

In the present study, decidualization process was observed dominantly in progesterone group. De Clercq *et al.* (2017) showed that deciualization was dependent to progesterone. This finding was aligned with the present study. Dursun *et al.* (2004) showed that exogenous gonadotropins could affect the morphology of endometrium and mitotic index during implantation, and these changes were more dominant in higher doses of exogenous gonadotropins.

The means of mast cell count were not significantly different between the groups neither in endometrium nor in myometrium. It means that the groups of study could not predict the mast cell count linearly. Tissue histamine level - as the clinical outcome of mast cell population - decreased in progesterone group in comparison to both control and induction groups. The mentioned findings mean that the effect of progesterone is *via* reduction in mast cell activity (i.e. histamine secretion).

According to the literature, the role of mast cells in implantation process and pregnancy is controversial. Although it has been known that estrogen and progesterone affect the population of mast cells (Noor *et al*., 2010; Wang *et al*., 2008), it seems that its increase may have different clinical outcomes in different conditions. In the present study, the authors found a non-significant increase in mast cell count of myometrium in induction and progesterone groups, and a non-significant decrease in mast cell count of endometrium in progesterone group. Hence, the authors intended to analyze the mast cell count ratio of myometrium per endometrium. Accordingly, this ratio was increased in progesterone group in comparison to control group based on Poisson regression. It seems that endometrial mast cells may play role against implantation while myometrium mast cells are necessary for better blood supply and uterine contractions. This interpretation should be studied specifically in future.

Historically, Harvey (1964) studied the distribution of mast cells in different days of estrous cycle in hamster. They found reduction of mast cells in endometrium and myometrium at the time of implantation. However, the percentage of myometrium mast cells per total mast cells increased (from 66% at day 1 to 96% at day 4) (Harvey, 1964). This result supported the hypothesis and result of the present study. Maraspin & Bo (1971) performed an animal study on a rat model. In their progesterone group, the mast cell count in myometrium per endometrium was 291 *versus* (*vs*.) 20 while in control group it was 255 *vs*. 25 and in estrogen group it was 150 *vs*. 31. They believed that mast cells play role in decidualization after pseudo pregnancy and therefore had considered positive effects for both endometrium and myometrium mast cell count (Maraspin & Bo, 1971). In the present study, there was no difference in this ratio between control and induction groups. The authors believe that after induction of ovulation decidualization is needed and progesterone help us to reach this aim. Mori *et al.* (1997) performed a human study. In their results, the mast cell count in inner myometrium per endometrium was 40 *vs*. 6 in proliferative phase while 30 vs 8 in secretory phase. Considering secretory phase as the time of decidualitation, the reduction of this ratio cannot be justified. However, it should be regarded that secretory phase has different physiology in pregnancy and non-pregnancy conditions. Bytautiene *et al.* (2004) found that myometrium mast cells and their degranulation could modulate uterine contractility during pregnancy. Szukiewicz *et al.* (2007) hypothesized that mast cell-derived interleukin-8 (IL-8) was associated with follicular growth and ovulation. They studied *in vitro* administration of IL-8 and found this association. It showed that the role of mast cells in female reproduction system was not limited to histamine secretion (Szukiewicz *et al*., 2007). Karaca *et al.* (2008) investigated the mast cells in female reproductive tract of goats. They found that distribution of the mast cells were different among the different parts of uterus in different days of the cycle (Karaca *et al*., 2008). Jensen *et al.* (2010) investigated the role of estradiol and progesterone in migration, maturation and degranulation of the mast cells in mice. They demonstrated that the hormones resulted in mast cell migration from periphery to uterus, maturation and degranulation as well as improve in angiogenesis (Jensen *et al*., 2010). Zierau *et al.* (2012) reviewed the role of estradiol and progesterone in mast cell behavior. They mentioned that female sex hormones induced mast cell maturation and degranulation (Zierau *et al*., 2012). Hamouzova *et al.* (2020) studied changes of mast cell distribution in canine uterus at early follicular and luteal phases of oestrous cycle. A significant decrease in mast cell count was observed at luteal phase in comparison to early follicular phase in both endometrium and myometrium. According to our calculation on their results, myo/endo ratio was 1.328 and 1.181 for luteal and early follicular phases respectively (Hamouzova *et al*., 2020).

The limitation of this study was lack of evaluating different parts of uterus separately (including horns, corpus and cervix), and releasing factors other than histamine. Although the authors had different results in comparison to the literature in terms of decrease in histamine level, it should be regarded that the present study had different design including pseudo pregnancy and induction of ovulation followed by progesterone administration. This result (reduction in histamine level based on the design of our study) may have beneficial effect for fertility of mice and possibly in human.

## CONCLUSIONS

Progesterone administration after induction of ovulation could not change the number of endometrial mast cells (ANOVA) and could increase the ratio of myometrium mast cells per endometrium mast cell (generalized linear model). Histamine level of uterus was decreased by administration of progesterone in ovulation induced mice. The ratio of mast cell in myometrium per endometrium is introduced to researchers for further study.
